# Supplementary material for: Content-rich biological network constructed by mining PubMed abstracts
Source: BMC Bioinformatics. 2004 Oct 8;5:147. doi: 10.1186/1471-2105-5-147 (PMC528731; doi:10.1186/1471-2105-5-147)
Supplement: Additional File 2 — The original results of the above study (non-essential files are deleted to keep the file size under the limit set by BMC bioinformatics). [file 1471-2105-5-147-S2.bz2 › chilibotAdditionalFile2/dip20/749ID10485884E18132/html/BTUB_TONB.html]

 


 **BTUB** and **TONB** 
  
Found 21 abstracts in PubMed, retrieved 20.  
 

 What does Google say? 
 PDF only 
| .edu only 

---

**Interactive relationship** (e.g. stimulation, inhibition, etc)

**Inhibitory relationship**- Mutations in  **tonB**  that suppressed the transport defect phenotype of these  **btuB**  mutations were isolated.  Ref: 2193917 J Bacteriol, 1990
- The various  **tonB**  mutations differed markedly in their suppression activities on different  **btuB**  or cir mutations.  Ref: 2193917 J Bacteriol, 1990
- A short sequence near the amino terminus of mature  **BtuB** , previously called the  **TonB**  box, is conserved in all  **tonB**  dependent receptors and colicins and is the site of the btuB451 mutation Leu 8 Pro, which prevents energy coupled cobalamin uptake.  Ref: 2687240 J Bacteriol, 1989
- This suppression activity was associated with the plasmid, suggesting that a mutation within the  **tonB**  gene on the plasmid allowed the mutant  **BtuB**  receptor to function in the transport of the vitamin.  Ref: 3294107 Gene, 1988
- This inhibition by CN Cbl is increased when the expression of FhuA and  **TonB**  is repressed by growth with excess iron and is eliminated when  **BtuB**  synthesis is repressed by CN Cbl.  Ref: 7665457 J Bacteriol, 1995
- The presence of the btuB451 mutation in this fusion gene eliminated all of its  **tonB**  coupled reactions, showing that the  **TonB**  box of FepA could be replaced by that from  **BtuB** .  Ref: 2687240 J Bacteriol, 1989
- Here, site directed disulfide crosslinking demonstrates contact between a conserved region of  **BtuB** , called the  **TonB**  box, and a portion of  **TonB** , previously implicated as the site of suppressors of  **TonB**  box mutations.  Ref: 10485884 Proc Natl Acad Sci U S A, 1999
- The Ton box sequence near the amino terminus of  **BtuB**  is conserved among all  **TonB**  dependent transporters and is the only known site of mutations that confer a transport defective phenotype which can be suppressed by certain substitutions at residue 160 in  **TonB** .  Ref: 11029413 J Bacteriol, 2000
**Neutral relationship**- Site directed disulfide bonding reveals an interaction site between energy coupling protein  **TonB**  and  **BtuB** , the outer membrane cobalamin transporter.  Ref: 10485884 Proc Natl Acad Sci U S A, 1999
- Energy coupled reactions of the Escherichia coli outer membrane transport proteins  **BtuB**  and Cir require the  **tonB**  product.  Ref: 2193917 J Bacteriol, 1990
- A short stretch of amino acids was homologous with the E. coli  **TonB**  dependent outer membrane proteins,  **BtuB** , IutA, FepA, and FhuA, suggesting a homologous function interaction with a periplasmic protein or uptake of a specific substrate.  Ref: 1885521 J Bacteriol, 1991
- Additional evidence for interaction of the Cbl and iron transport systems is provided by the strong stimulation of the  **BtuB**  and  **TonB**  dependent transport of CN Cbl into a nonexchangeable, presumably cytoplasmic pool by preincubation of cells with the iron II chelator dipyridyl.  Ref: 7665457 J Bacteriol, 1995
- Sequence changes in the ton box region of  **BtuB**  affect its transport activities and interaction with  **TonB**  protein.  Ref: 11029413 J Bacteriol, 2000
- Some point mutations in a region of  **btuB**  and cir that is highly conserved in  **TonB**  dependent transport proteins led to loss of  **TonB**  coupled uptake of vitamin B12 and colicin Ia.  Ref: 2193917 J Bacteriol, 1990
- The Escherichia coli  **btuB**  product is an outer membrane protein that mediates the  **TonB**  coupled active transport of cobalamins and the uptake of the E colicins and bacteriophage BF23.  Ref: 1885541 J Bacteriol, 1991
- This interaction with  **TonB**  occurs in part through an N terminal segment in the  **BtuB**  sequence called the Ton box.  Ref: 11705387 Biochemistry, 2001
- Uptake of cobalamins by the transporter protein  **BtuB**  in the outer membrane of Escherichia coli requires the proton motive force and the transperiplasmic protein  **TonB** .  Ref: 11029413 J Bacteriol, 2000
- The bacterial outer membrane transporter for vitamin B 12,  **BtuB** , derives its energy for transport by interacting with the trans periplasmic membrane protein  **TonB** .  Ref: 11705387 Biochemistry, 2001
- Cobalamin Cbl transport across the outer membrane of cells of Escherichia coli consists of high affinity Cbl binding to the  **btuB**  protein of the Cbl receptor, followed by the proton motive force and  **tonB**  dependent release of the Cbl into the periplasmic space.  Ref: 3512539 J Biol Chem, 1986
- These data are summarized on a two dimensional folding model for the  **BtuB**  protein in the outer membrane devised using both amphipathic beta strand analysis and sequence conservation amongst the  **TonB**  dependent receptors.  Ref: 7746157 Mol Microbiol, 1995
- The uncoupled proline substitutions in  **BtuB**  markedly affected the pattern of disulfide bonding to  **TonB** , both increasing the extent of cross linking and shifting the pairs of residues that can be joined.  Ref: 11029413 J Bacteriol, 2000
- Site directed disulfide bonding in intact cells has been used to show that the Ton box of  **BtuB**  and residues around position 160 of  **TonB**  approach each other in a highly oriented and specific manner to form  **BtuB**   **TonB**  heterodimers that are stimulated by the presence of transport substrate.  Ref: 11029413 J Bacteriol, 2000

**Non-interactive relationship** (e.g. studied together, co-existance, homology, etc.)

- Transport of vitamin B 12 across the outer membrane of Escherichia coli, like that of iron siderophore complexes, is an active transport process requiring a specific outer membrane transporter  **BtuB** , the proton motive force, and the trans periplasmic energy coupling protein  **TonB** .  Ref: 10485884 Proc Natl Acad Sci U S A, 1999
- Cross linking of  **BtuB**  and  **TonB**  in the presence of the  **BtuB**  V10P substitution became independent of the presence of substrate, indicating an additional distortion of the exposure of the Ton box in the periplasmic space.  Ref: 11029413 J Bacteriol, 2000
- FyuA shows homology to other iron regulated  **TonB**  dependent outer membrane proteins with receptor functions e.g.  **BtuB** , CirA, FepA, IutA, FhuA, FoxA, FcuA.  Ref: 7984105 Mol Microbiol, 1994
- The defect in cobalamin utilization resulting from the L8P substitution was suppressed by cysteine substitutions in adjacent residues in  **BtuB**  or in  **TonB** .  Ref: 11029413 J Bacteriol, 2000
- We consider a variety of different colicins, including A, B, D, E1, E3, Ia, M and N, that penetrate through the porins OmpF, FepA,  **BtuB** , Cir and FhuA, to subsequently interact with a few targets in the periplasm, including TolA, TolB, TolC and  **TonB** .  Ref: 12423783 Biochimie< MedlineTA>Biochimie, 2000
- Comparison of the deduced amino acid sequence of FepA with that of three other  **TonB**  dependent receptors  **BtuB** , FhuA, and IutA revealed only a few regions of sequence homology.  Ref: 3015941 J Biol Chem, 1986
- In addition to this plasmid with a 6 kilobase insert of S. typhimurium DNA, vitamin B12 utilization by E. coli  **btuB**  strains required the btuC and btuD products, necessary for transport across the cytoplasmic membrane, but not the btuE or  **tonB**  product.  Ref: 2656634 J Bacteriol, 1989
- These results suggest that the  **TonB**  box region of  **BtuB**  is involved in active transport in a manner dependent not on the identity of specific side chains but on the local secondary structure.  Ref: 2687240 J Bacteriol, 1989
- Transport of CN Cbl across the outer membrane and its accumulation in the periplasm is mediated by the  **TonB**  dependent transporter  **BtuB** .  Ref: 11790740 J Bacteriol, 2002
- Point mutations in a conserved region  **TonB**  box of Escherichia coli outer membrane protein  **BtuB**  affect vitamin B12 transport.  Ref: 2687240 J Bacteriol, 1989
- Crosslinking at several positions was increased when  **BtuB**  was loaded with substrate, and the crosslinking pattern was altered by the presence of substitutions in  **BtuB**  that cause a  **TonB**  uncoupled phenotype.  Ref: 10485884 Proc Natl Acad Sci U S A, 1999
- In a transport defective mutant of  **BtuB** , this conformational cycle is disrupted and the  **TonB**  box appears to be extended even in the absence of substrate.  Ref: 10700278 Nat Struct Biol, 2000
- To examine the role of individual amino acids in the  **TonB**  box of  **BtuB** , more than 30 amino acid substitutions in residues 6 to 13 were generated by doped oligonucleotide directed mutagenesis.  Ref: 2687240 J Bacteriol, 1989
